# Heat-Inactivated *Bifidobacterium longum* Z1 Improves Recognition Memory in Mice in Association with Enhanced Trk-CREB Signaling and Reduced Methylglyoxal Levels

**DOI:** 10.3390/ijms272110054

**Published:** 2026-11-09

**Authors:** Seong-Min Hong, Jaehoon Bae, Seojin Kang, Hyunjin Kang, Dong-Hyun Kim, Sun Yeou Kim

**Affiliations:** 1 Division of Food Science and Technology and Institute of Agriculture and Life Science, Gyeongsang National University, Jinju-si 52828, Republic of Korea; hsm0206@gnu.ac.kr (S.-M.H.); kseojin@gnu.ac.kr (S.K.); endrmfdnjs@gnu.ac.kr (H.K.); 2 Functional Food Research Institute, Industry-University Cooperation Foundation, Daegu Haany University, Gyeongsan-si 38610, Republic of Korea; baejaehoon@dhu.ac.kr; 3 Department of Food Industry, Daegu Haany University, Gyeongsan-si 38610, Republic of Korea; 4 Neurobiota Research Center, College of Pharmacy, Kyung Hee University, Seoul 02447, Republic of Korea; 5 College of Pharmacy and Gachon Institute of Pharmaceutical Science, Gachon University, Incheon 21936, Republic of Korea

**Keywords:** *Bifidobacterium longum* Z1, postbiotics, psychobiotics, microbiota–gut–brain axis, recognition memory, neurotrophin, Trk receptors, CREB, Nrf-2, methylglyoxal

## Abstract

Probiotics can modulate brain function via the microbiota–gut–brain axis; however, their cognitive effects in healthy hosts and the underlying pathways remain unclear. We previously reported that *Bifidobacterium longum* Z1 protects neuronal cells against methylglyoxal (MGO)-induced glycotoxicity. Here, we investigated whether oral heat-inactivated *B. longum* Z1 enhances memory in normal mice and examined associated molecular changes. Male C57BL/6 mice received heat-inactivated *B. longum* Z1 (5 or 20 mg/kg/day) or piracetam (400 mg/kg/day) for seven days before behavioral testing. High-dose *B. longum* Z1 (High-Z1) significantly increased the recognition index in the novel object recognition test. In exploratory brain analyses of mice selected by recognition index, High-Z1 increased the level of a nerve growth factor-immunoreactive band and the phosphorylation of tropomyosin receptor kinase B (TrkB) and cAMP response element-binding protein (CREB), whereas TrkA phosphorylation was not significantly changed. High-Z1 reduced MGO levels in the liver, brain, and serum, and in a separate exploratory cohort, a higher dose (100 mg/kg) upregulated hepatic glyoxalase-I and II and increased nuclear levels of nuclear factor erythroid 2-related factor-2 (Nrf-2). These molecular changes are associative and do not establish that neurotrophin-Trk-CREB signaling, the glyoxalase pathway, or MGO reduction mediates the behavioral effect. These findings support further investigation of this heat-inactivated *B. longum* Z1 preparation as a postbiotic for cognitive health.

## 1. Introduction

Cognitive decline, ranging from subtle age-associated memory loss to dementia, is a growing public health concern. Strategies that support cognitive function in otherwise healthy individuals are of considerable interest. Over the past two decades, the gut microbiota has emerged as a key regulator of brain function through the microbiota–gut–brain axis, a bidirectional communication network involving immune signaling, microbial metabolites, the enteric nervous system, and the vagus nerve [1,2,3].

Among gut commensals, members of the genus *Bifidobacterium* have been widely studied as “psychobiotics,” that is, microorganisms with potential mental health benefits. *B. longum* 1714 improved object recognition and spatial learning in mice [4] and produced subtle improvements in hippocampus-dependent visuospatial memory in healthy volunteers [5]. *B. breve* A1 prevented cognitive dysfunction in an amyloid-β-induced Alzheimer’s disease mouse model [6] and improved cognitive scores in older adults with mild cognitive impairment [7]. *B. longum* NK46 alleviated cognitive decline and increased hippocampal brain-derived neurotrophic factor (BDNF) expression in 5XFAD transgenic and aged mice [8]. Similarly, *B. longum* NCC3001 normalized anxiety-like behavior and hippocampal BDNF levels in mice with colitis [9]. Importantly, these effects are highly strain-specific [4], underscoring the need for individual characterization of each candidate strain.

Neurotrophins are central mediators of synaptic plasticity and memory [10,11]. Nerve growth factor (NGF) and BDNF act through their high-affinity receptors, tropomyosin receptor kinase A (TrkA) and TrkB, respectively, activating downstream phosphatidylinositol 3-kinase (PI3K)/AKT and mitogen-activated protein kinase (MAPK)/extracellular signal-regulated kinase (ERK) cascades [12]. These pathways converge on the transcription factor cAMP response element-binding protein (CREB), whose phosphorylation is required for the conversion of short-term to long-term memory and, consequently, drives BDNF transcription [13,14].

Methylglyoxal (MGO) is a highly reactive dicarbonyl formed mainly as a by-product of glycolysis and a major precursor of advanced glycation end products [15]. MGO is detoxified by the glyoxalase system, in which glyoxalase-I (Glo-1) converts MGO-glutathione hemithioacetal into *S*-D-lactoylglutathione, which is subsequently hydrolyzed to D-lactate by glyoxalase-II (Glo-2) [16,17]. Accumulation of MGO, termed dicarbonyl stress, has been implicated in aging and age-related disorders [17]. In older adults, higher circulating MGO is associated with faster cognitive decline, poorer memory, and lower grey matter volume [18,19]; MGO reduces the expression of neurotrophic factors in neuronal cells [20]. Moreover, altered Glo-1 expression and activity have been reported in the brains of individuals with aging and Alzheimer’s disease [21,22]. In our previous study, we found that MGO administration impaired memory and reduced the expression of BDNF, NGF, and phosphorylated TrkB, together with the suppression of nuclear factor erythroid 2-related factor 2 (Nrf-2) signaling, whereas tryptophan supplementation reversed these changes and upregulated the expression of Glo-1 and Glo-2 [15]. Similarly, chronic oral MGO exposure induces Alzheimer-like cognitive deficits, hippocampal neuronal damage, and suppression of PI3K/AKT signaling in mice [23]. Notably, Nrf-2 binds to a functional antioxidant response element (ARE) in the *GLO1* gene [24], linking antioxidant defenses to dicarbonyl detoxification. Thus, lowering the MGO burden by, for example, activating the Nrf-2-glyoxalase pathway is a plausible strategy for preserving neurotrophic signaling and maintaining neuronal function.

We recently isolated *B. longum* Z1 from human feces and showed that it protects mouse neuroblastoma (N2a) cells against MGO-induced apoptosis, oxidative stress, and inflammatory signaling by enhancing glyoxalase system activity and Nrf-2-mediated antioxidant defenses [25]. Untargeted metabolite profiling of *B. longum* Z1 cells further identified 47 metabolites, including 25 amino acids and their derivatives; among the amino acids abundant in *B. longum* Z1, tryptophan exhibited the highest affinity for MGO and protected N2a cells against MGO-induced toxicity [25]. Notably, *B. longum* Z1, used as heat-inactivated cells, can be classified as a postbiotic and is defined as a preparation of inactivated microorganisms and/or their components that provides health benefits to the host [26]. For example, dietary heat-killed *Lactobacillus brevis* SBC8803 improved hippocampus-dependent memory and adult hippocampal neurogenesis in mice [27], indicating that live bacteria are not always required for cognitive benefits. Because gut-derived microbial products and metabolites reach the liver first through the portal circulation [28,29], orally administered *B. longum* Z1 may also enhance hepatic MGO detoxification, thereby reducing the MGO burden reaching the brain. However, whether *B. longum* Z1 affects cognitive function in vivo has not been determined. Therefore, we hypothesized that oral *B. longum* Z1 would improve memory in normal mice by enhancing neurotrophin-Trk-CREB signaling in the brain and activating the Nrf-2-glyoxalase pathway in the liver. To test this hypothesis, we examined recognition memory using piracetam as a reference nootropic [30] and evaluated brain neurotrophic signaling, hippocampal histology, tissue MGO levels, hepatic Nrf-2-glyoxalase signaling, and serum liver enzymes and hepatic and intestinal histology after *B. longum* Z1 administration.

## 2. Results

### 2.1. B. longum Z1 Improved Recognition Memory in the Novel Object Recognition Test

**Figure 1:**
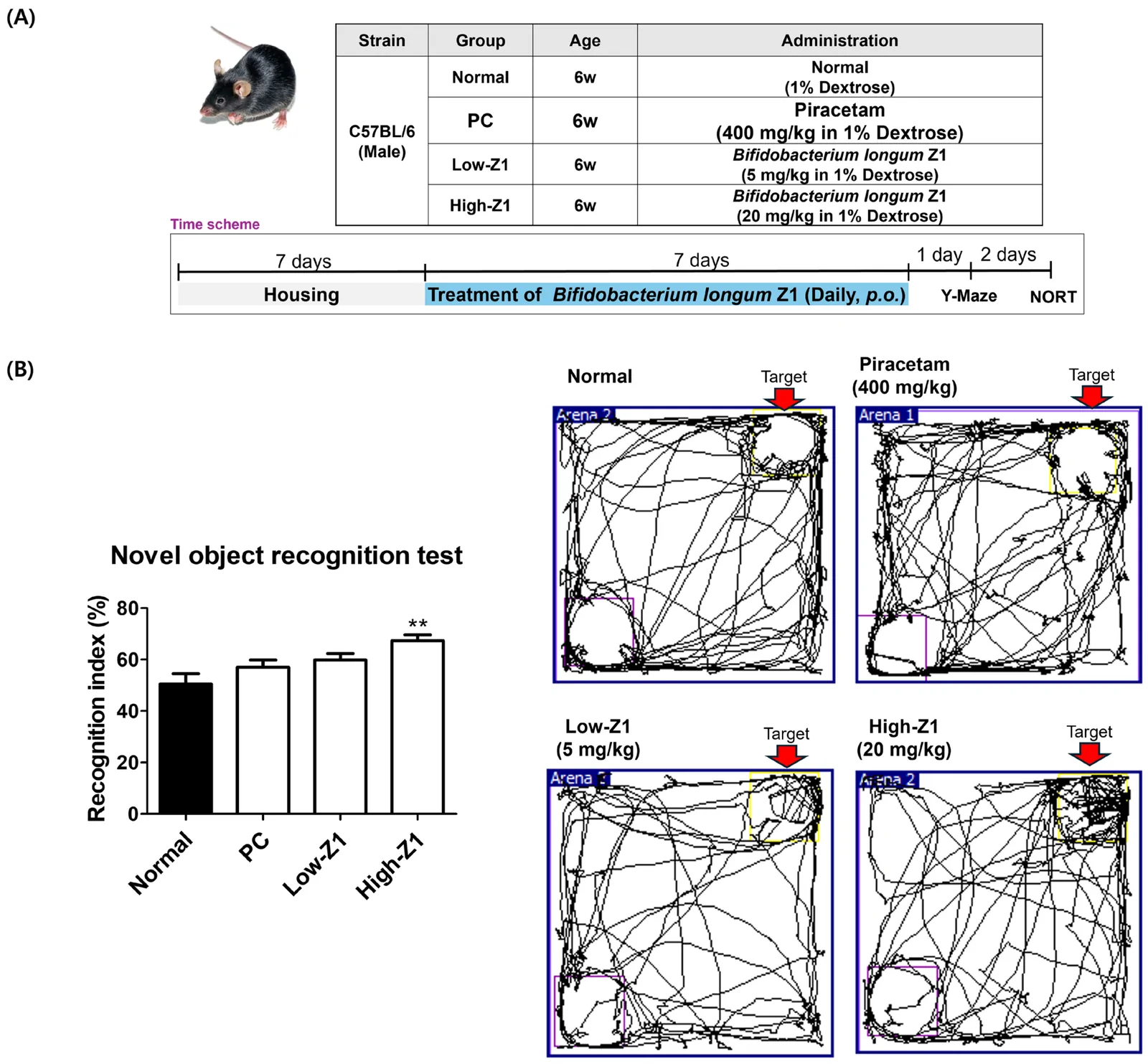
Experimental scheme and the effect of *Bifidobacterium longum* Z1 on recognition memory in mice. (**A**) Schematic representation of animal experimental grouping and treatment timeline. Male C57BL/6 mice (6 weeks old) were divided into four groups: Normal (1% dextrose), PC (positive control, piracetam 400 mg/kg), Low-Z1 (5 mg/kg), and High-Z1 (20 mg/kg). Treatments were orally administered daily for seven days, followed by behavioral assessments including the Y-maze test (data not reported; see Section 4.3) and novel object recognition test (NORT). (**B**) Recognition index (%) and representative exploration track heatmaps in the NORT. Data are presented as mean ± standard error of the mean (SEM) (*n* = 7 per group). ** *p* < 0.01 vs. Normal group (one-way ANOVA with Tukey’s post hoc test). Representative exploration track heatmaps were generated with SMART 3.0. Individual data, including the original analysis after post hoc exclusion of one animal per group, are provided in Supplementary Table S2, and object exploration times are provided in Supplementary Table S4.

To evaluate the effect of *B. longum* Z1 on cognitive function under physiological conditions, normal male C57BL/6 mice were orally administered 1% dextrose (Normal), piracetam (400 mg/kg; positive control [PC]), or heat-inactivated *B. longum* Z1 at 5 mg/kg (Low-Z1) or 20 mg/kg (High-Z1) daily for seven days, followed by the novel object recognition test (NORT) (Figure 1A). In the NORT, the High-Z1 group (67.39 ± 2.25%, ** *p* < 0.01) showed a significantly higher recognition index than that of the Normal group (50.42 ± 4.05%), whereas the increases in the Low-Z1 and PC groups did not reach statistical significance (Figure 1B). Consistent with this finding, representative exploration tracks showed denser trajectories around the novel (target) object in High-Z1 mice than in normal mice (Figure 1B). All available animals were included in this analysis (*n* = 7 per group); the result was unchanged in the original analysis after post hoc exclusion of one animal per group (*n* = 6 per group; *p* = 0.007; Supplementary Table S2). Total exploration time did not differ among groups during training or testing, and no side preference was observed during training (Supplementary Table S4). The Low-Z1 and High-Z1 groups explored the novel object significantly longer than the familiar object, whereas the Normal and PC groups did not show a significant preference. The higher recognition index in the High-Z1 group was associated mainly with reduced exploration of the familiar object.

### 2.2. Neurotrophin-Related Proteins and Trk-CREB Signaling in the Brain After B. longum Z1 Administration (Exploratory Analysis)

**Figure 2:**
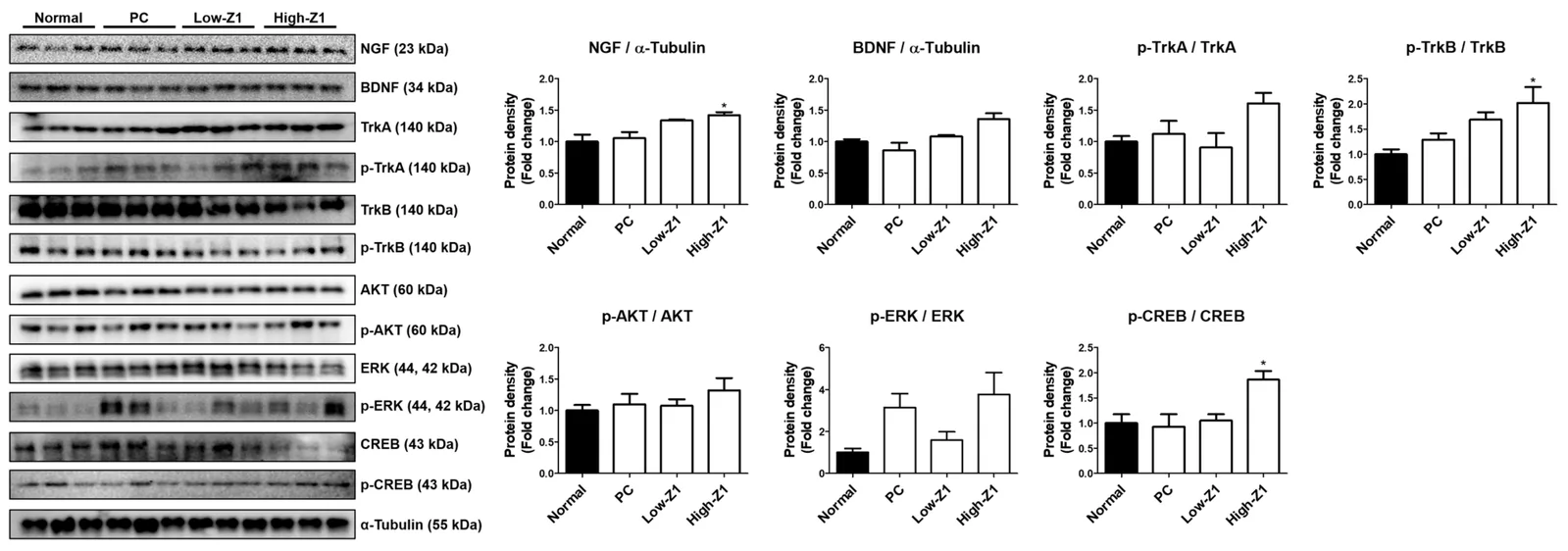
Effect of *B. longum* Z1 on neurotrophic signaling in brain-hemisphere lysates. Representative Western blots and densitometric quantification of NGF, BDNF, TrkA, phospho(p)-TrkA, TrkB, p-TrkB, AKT, p-AKT, ERK, p-ERK, CREB, and p-CREB. The NGF- and BDNF-immunoreactive bands (approximately 23 and 34 kDa) were normalized to α-tubulin and phosphorylated proteins to their total forms, each detected on a separate gel loaded with the same lysates in the same lane order. Protein levels are expressed as fold change relative to the Normal group. Data are presented as mean ± SEM (*n* = 3 per group; animals selected by NORT performance). * *p* < 0.05 vs. Normal group (one-way ANOVA with Tukey’s post hoc test).

To examine molecular changes associated with the improved recognition memory, we analyzed neurotrophin signaling in brain-hemisphere lysates using Western blotting (Figure 2). For these analyses, the three mice per group with the highest NORT recognition index were selected after behavioral testing (Section 4.5); the molecular data therefore describe this subset rather than the whole group and are exploratory. High-Z1 significantly increased the level of the NGF-immunoreactive band (approximately 23 kDa; 1.42-fold, * *p* < 0.05), TrkB phosphorylation (2.02-fold, * *p* < 0.05), and CREB phosphorylation (1.86-fold, * *p* < 0.05) compared with the Normal group. BDNF expression and the phosphorylation of TrkA, AKT, and ERK also tended to increase in the High-Z1 group, although these changes were not statistically significant and should be regarded as hypothesis-generating; for example, TrkA phosphorylation in the High-Z1 group was 1.61-fold that in the Normal group (*p* = 0.161). TrkB phosphorylation in the Low-Z1 group was intermediate between that in the Normal and High-Z1 groups but did not differ significantly from the Normal group. Piracetam did not significantly alter any of the proteins examined; ERK phosphorylation was numerically higher (3.14-fold), but the difference was not significant.

### 2.3. Granule Cell Count per Field in the Hippocampal Dentate Gyrus After B. longum Z1 Administration

**Figure 3:**
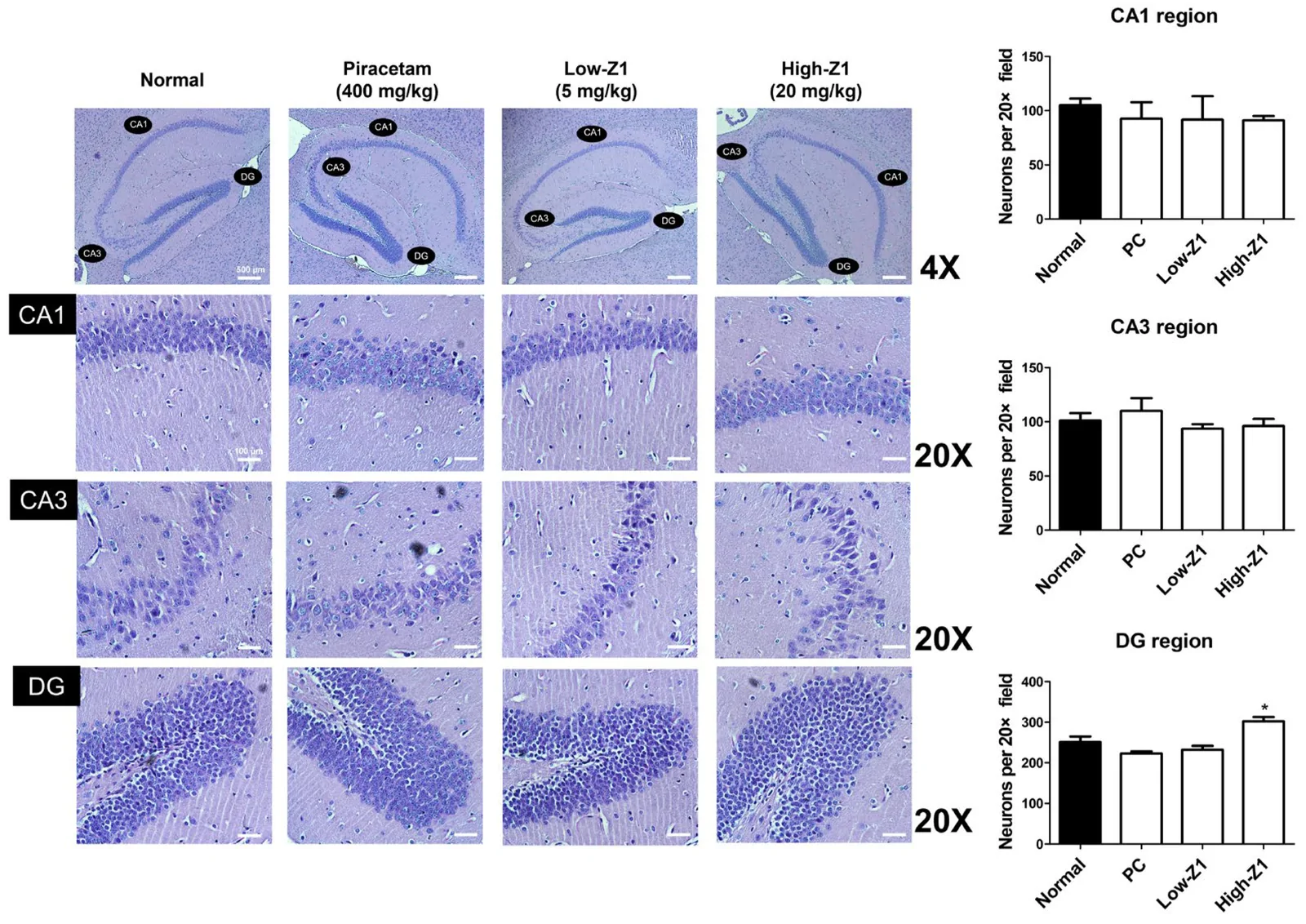
Histological analysis and neuronal counts per field in the hippocampal regions CA1, CA3, and DG. Representative hematoxylin and eosin-stained coronal hippocampal sections at low (4×; scale bar, 500 μm) and high (20×; scale bar, 100 μm) magnification, showing the cornu ammonis (CA) 1, CA3, and dentate gyrus (DG) subregions, with corresponding quantification of neuronal counts per 20× field (mean of three fields in one section per animal; values do not represent total neuronal number). Data are presented as mean ± SEM (*n* = 3). * *p* < 0.05 vs. Normal group (one-way ANOVA with Tukey’s post hoc test).

The hippocampal morphology was examined in H&E-stained coronal sections (Figure 3). Histology was performed on the same three mice per group used for Western blotting, selected by NORT performance (Section 4.6); these counts are therefore exploratory. No apparent neuronal loss, pyknosis, or structural disorganization was observed in the CA1 and CA3 subfields of the cornu ammonis (CA) or the dentate gyrus (DG) in any group. Quantitative analysis showed that the neuronal counts per field in CA1 and CA3 did not differ among the groups. In contrast, the neuronal count per field in the DG granule cell layer was significantly higher in the High-Z1 group (302 cells/field) than in the Normal group (251 cells/field; * *p* < 0.05), whereas the counts in the Low-Z1 and PC groups were comparable to those in the Normal group (Figure 3). These values represent counts per 20× field and do not indicate the total number of neurons in the DG.

### 2.4. Methylglyoxal Levels in the Liver, Brain, and Serum After B. longum Z1 Administration

**Figure 4:**
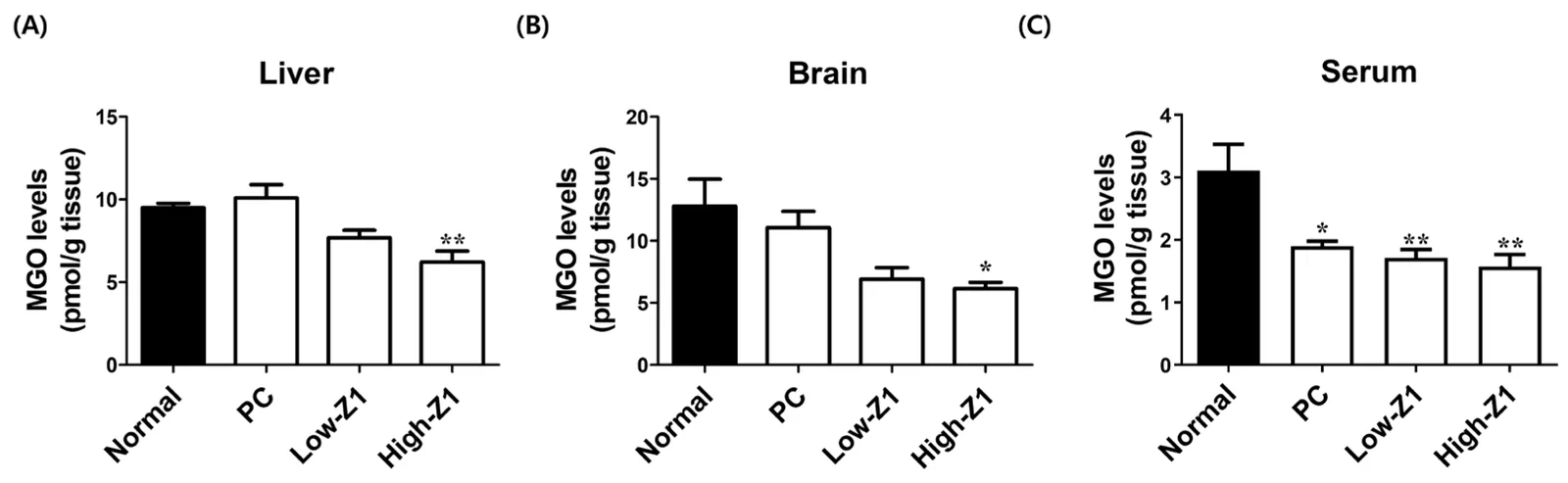
Effects of *B. longum* Z1 on methylglyoxal (MGO) levels in the (**A**) liver, (**B**) brain, and (**C**) serum. MGO concentrations were determined by HPLC after *o*-phenylenediamine derivatization. Data are presented as mean ± SEM (*n* = 6 for liver and serum; *n* = 4 for brain). * *p* < 0.05 and ** *p* < 0.01 vs. Normal group (one-way ANOVA with Tukey’s post hoc test).

Given our previous finding that B. longum Z1 enhances MGO detoxification in vitro [25], we measured MGO concentrations in the liver, brain, and serum using high-performance liquid chromatography (HPLC) (Figure 4). Liver and serum MGO were measured in the six mice per group whose tissues had been processed, and brain MGO in the four mice per group with the highest NORT recognition index (Section 4.8); the brain data are therefore exploratory. In the liver, MGO levels were decreased by 19.2% and 34.6% in the Low-Z1 (7.68 ± 0.47 pmol/g tissue) and High-Z1 (6.21 ± 0.67 pmol/g tissue; ** *p* < 0.01) groups, respectively, compared with the Normal group (9.51 ± 0.26 pmol/g tissue); only the decrease in the High-Z1 group was significant, and piracetam had no significant effect (10.10 ± 0.80 pmol/g tissue). In the brain, MGO levels were reduced by 46.0% in the Low-Z1 group (6.91 ± 0.93 pmol/g tissue) and 52.0% in the High-Z1 group (6.14 ± 0.52 pmol/g tissue; * *p* < 0.05) compared with the Normal group (12.79 ± 2.19 pmol/g tissue), whereas piracetam exhibited a non-significant reduction of 13.5% (11.05 ± 1.32 pmol/g tissue). In serum, MGO levels were significantly lower in all treated groups than in the Normal group (3.11 ± 0.42 pmol/mL), with reductions of 38.9% in the PC group (1.90 ± 0.08 pmol/mL; * *p* < 0.05), 44.7% in the Low-Z1 group (1.72 ± 0.13 pmol/mL; ** *p* < 0.01), and 49.3% in the High-Z1 group (1.58 ± 0.19 pmol/mL; ** *p* < 0.01). Thus, High-Z1 significantly decreased MGO levels in all three matrices, whereas the smaller reductions in the Low-Z1 group were significant only in serum.

### 2.5. Hepatic Nrf-2 and Glyoxalase Signaling After a Higher Dose of B. longum Z1 in a Separate Cohort

To examine whether *B. longum* Z1 can activate the hepatic glyoxalase system in vivo, we performed a separate exploratory experiment in which mice received a higher dose of *B. longum* Z1 (100 mg/kg; *n* = 5) or vehicle (*n* = 4) for seven days (Figure S1). A higher dose than in the main study was used to increase the likelihood of detecting a pathway response in healthy mice. *B. longum* Z1 significantly increased the expression of Glo-1 (approximately 1.35-fold; * *p* < 0.05) and Glo-2 (approximately 1.5-fold; ** *p* < 0.01). Because Glo-1 transcription is regulated by Nrf-2 through an ARE [24], we also examined the Nrf-2 pathway. Nuclear Nrf-2 levels were significantly increased (approximately 1.5-fold; * *p* < 0.05), whereas cytosolic Nrf-2 and Kelch-like ECH-associated protein 1 (Keap1) levels were only slightly increased. Heme oxygenase-1 (HO-1) showed an increasing trend, although the change did not reach statistical significance. Because hepatic Nrf-2/glyoxalase signaling is reported only for this separate cohort, these data do not establish whether this pathway is activated at 5 or 20 mg/kg.

### 2.6. Serum Liver Enzymes and Hepatic and Intestinal Histology After Short-Term Administration of B. longum Z1

**Figure 5:**
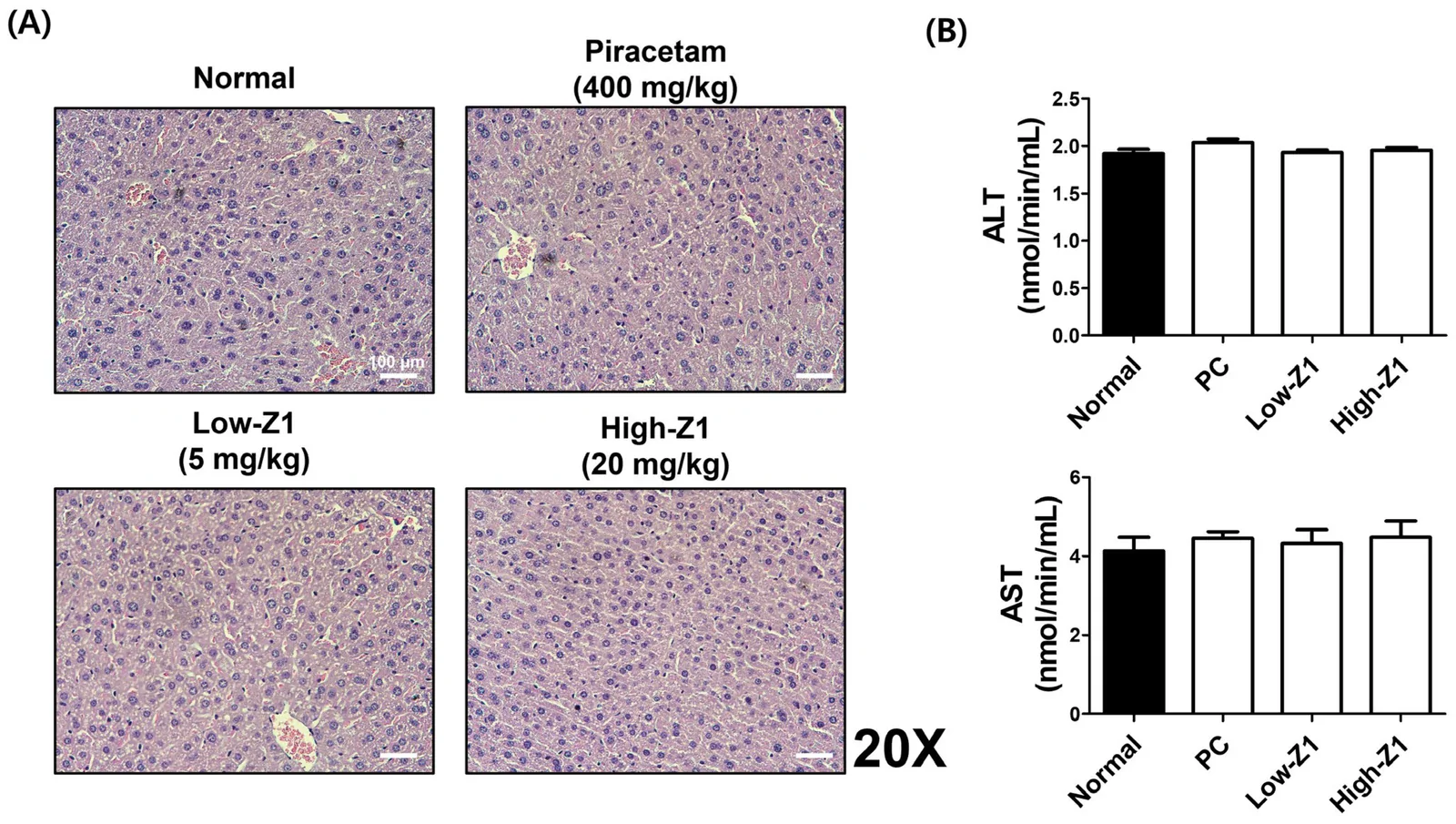
Hepatic histology and serum liver enzymes after *B. longum* Z1 administration. (**A**) Representative H&E-stained liver sections (20×; scale bar, 100 μm). (**B**) Serum ALT and AST activities. Data are presented as mean ± SEM (*n* = 6); no significant differences were observed among the groups (one-way ANOVA with Tukey’s post hoc test).

**Figure 6:**
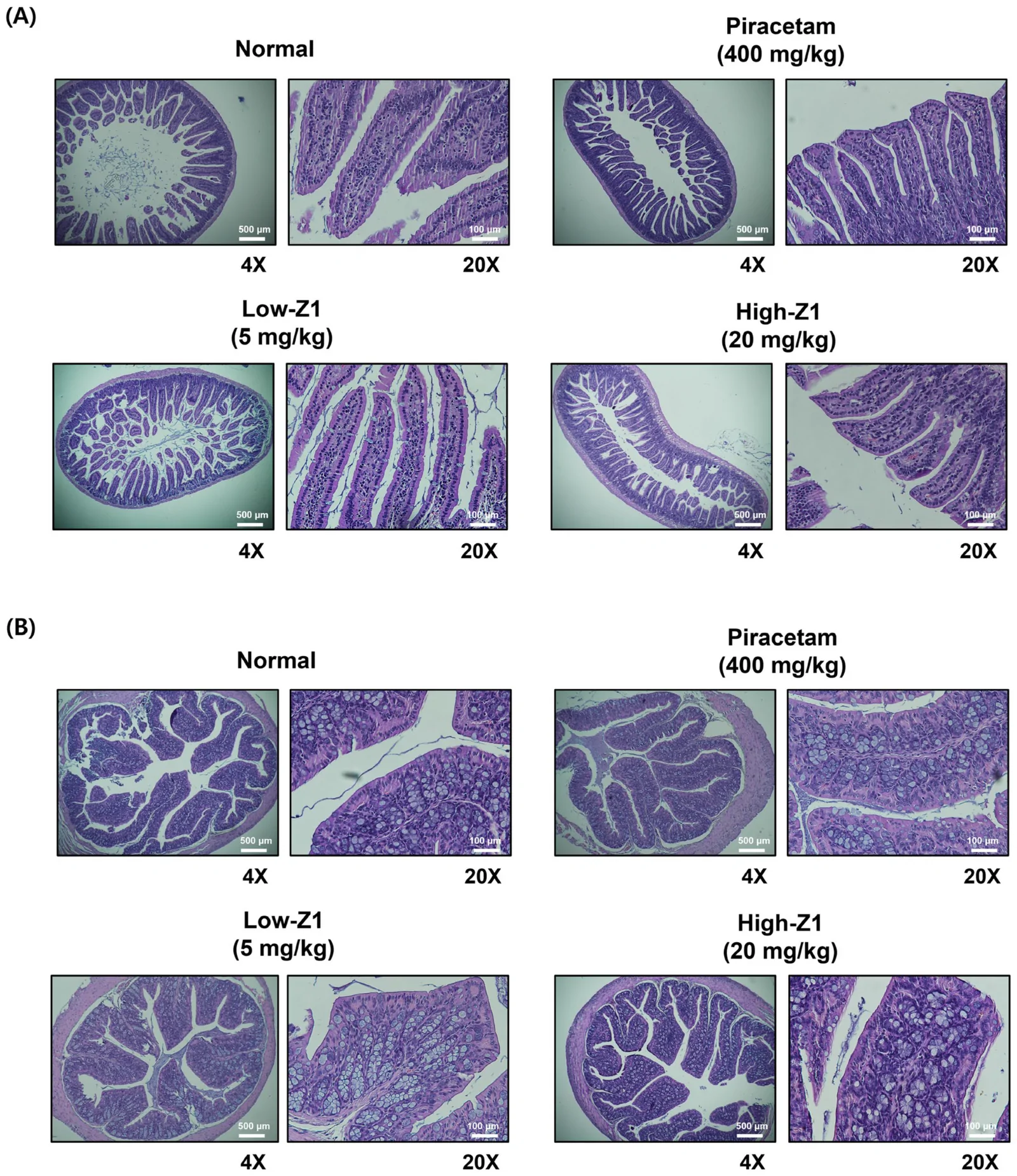
Intestinal histology after *B. longum* Z1 administration. Representative H&E-stained sections of the (**A**) small intestine and (**B**) large intestine at low (4×; scale bar, 500 μm) and high (20×; scale bar, 100 μm) magnification.

Because the liver and intestine are the primary organs exposed to orally administered bacterial preparations, we examined serum liver enzymes and hepatic and intestinal histology after *B. longum* Z1 administration. Liver sections from all groups exhibited normal hepatic architecture with regularly arranged hepatocyte cords and no evidence of inflammatory cell infiltration, necrosis, or steatosis (Figure 5A). Serum alanine aminotransferase (ALT) and aspartate aminotransferase (AST) levels did not differ significantly among the groups (Figure 5B). Similarly, the small and large intestines of all groups showed intact mucosal structures, including well-preserved villi, crypts, and goblet cells, without erosion or inflammatory infiltration (Figure 6). Thus, no significant changes in serum ALT or AST and no apparent histological abnormalities in the liver or intestine were observed after 7 days of administration at 5 or 20 mg/kg.

## 3. Discussion

In this study, we found that short-term oral administration of heat-inactivated *B. longum* Z1 improved recognition memory in normal mice. In exploratory analyses of mice selected by NORT performance, this behavioral effect was accompanied by higher levels of an NGF-immunoreactive band and TrkB and CREB phosphorylation in the brain and a higher neuronal count per field in the hippocampal DG; MGO levels were lower in the liver, brain, and serum. In a separate exploratory cohort, a higher dose (100 mg/kg) upregulated hepatic Glo-1 and Glo-2 expression and increased nuclear Nrf-2 levels. No significant changes in serum liver enzymes or apparent hepatic or intestinal histological abnormalities were observed under the conditions tested. To our knowledge, this is the first in vivo study to evaluate the cognitive effects of *B. longum* Z1.

The improvement in NORT performance in healthy mice is consistent with previous reports showing that *B. longum* 1714 enhances object recognition in mice without any prior challenge [4] and modestly improves hippocampus-dependent memory in healthy humans [5]. Total object exploration in the NORT was comparable among groups, suggesting that the NORT result was not driven by a general change in activity. However, the higher recognition index in the High-Z1 group reflected mainly reduced exploration of the familiar object, so a contribution of non-mnemonic factors, such as altered novelty seeking or motivation, cannot be ruled out. Notably, piracetam did not significantly improve performance in the NORT. The clinical efficacy of piracetam has been documented mainly in cognitive disorders [30]; therefore, its effect in cognitively intact young animals may be limited at the dose and duration used here. Because the reference compound did not produce a significant effect, the assay sensitivity of the behavioral design was not confirmed by a positive control in this setting; this limits the interpretation of the behavioral data, and the effect of *B. longum* Z1 should be confirmed in models of cognitive impairment with a validated positive control. Because High-Z1 and piracetam did not differ significantly in a direct comparison, these data do not indicate that *B. longum* Z1 is more effective than piracetam.

At the molecular level, High-Z1 was associated with higher levels of the NGF-immunoreactive band and higher TrkB and CREB phosphorylation; because these analyses were performed in mice selected by NORT performance, they are exploratory and cannot establish the mechanism of the behavioral effect. TrkB signaling is a potent regulator of hippocampal long-term potentiation and learning [14], and CREB-dependent transcription is required for memory consolidation [13]. Since CREB also drives BDNF transcription, the concurrent increase in p-CREB and the upward trend in BDNF would be consistent with a positive feedback loop, although this was not tested. NGF/TrkA signaling promotes neuronal survival and neurite outgrowth via the PI3K/AKT and MAPK/ERK pathways [10]. These findings are consistent with those of previous studies showing that *Bifidobacterium* strains increase hippocampal BDNF expression in mouse models of colitis, Alzheimer’s disease, and aging [6,8,9]. However, we analyzed brain-hemisphere lysates, which may have diluted region-specific changes. This may explain why several downstream effectors (p-AKT and p-ERK) showed only non-significant trends. Hippocampal and cortex-specific analyses in randomly selected animals are required to determine whether these changes occur in specific brain regions.

High-Z1 was also associated with a higher neuronal count per field in the DG, but not in the CA1 or CA3 regions. Because only one H&E-stained section per animal was analyzed, without stereological sampling or blinding, in animals selected by NORT performance, this finding is exploratory and indicates only a higher granule cell count per field. It does not show a change in total DG neuron number and may reflect differences in cell packing or sampling position, and H&E staining cannot distinguish newborn from mature neurons, so these data provide no information on adult neurogenesis, which would require markers of newborn neurons and stereological counting [31].

A distinctive finding of this study was that High-Z1 reduced MGO levels not only in the liver but also in the brain and serum. In a separate cohort, a higher dose of B. longum Z1 (100 mg/kg) increased hepatic Glo-1 and Glo-2 protein levels together with nuclear Nrf-2 levels, suggesting that B. longum Z1 can upregulate both enzymes of the glyoxalase pathway, which converts MGO into D-lactate. Because this pathway is reported only for the 100 mg/kg cohort, the present data do not establish that it contributes to the MGO reduction at 5 and 20 mg/kg, and this reduction cannot be attributed to glyoxalase induction. Whether glyoxalase induction contributes to the MGO-lowering effect at lower doses remains to be determined. Nrf-2 binds to a functional ARE in the *GLO1* gene, and Glo-1 expression is decreased in the liver and brain of Nrf-2-deficient mice [24], suggesting that Nrf-2 activation is a plausible upstream mechanism for Glo-1 induction. These in vivo results are consistent with our previous finding that *B. longum* Z1 activates the glyoxalase system and Nrf-2/HO-1 signaling in MGO-challenged neuronal cells [25]. Under basal conditions, Keap1 retains Nrf-2 in the cytoplasm and promotes its degradation, whereas electrophilic or oxidative signals release Nrf-2 from Keap1-mediated repression [32]. Therefore, the modest increase in Keap1 expression observed at 100 mg/kg may reflect a feedback response of the Keap1-Nrf-2 pathway following Nrf-2 activation, although the upstream signal that triggers Nrf-2 activation by *B. longum* Z1 remains unknown. At the lower doses used in the behavioral study, other mechanisms may also be involved. Our previous metabolite profiling of *B. longum* Z1 cells identified 47 metabolites, with tryptophan showing the highest affinity for MGO among the 13 amino acids tested [25]. Because the heat-inactivated *B. longum* Z1 preparation used in this study contained these intracellular metabolites, direct trapping of MGO by tryptophan and other bacterial metabolites may further contribute to the reduction in systemic MGO, although residual medium constituents in the unwashed preparation may also have contributed. In particular, MGO exposure is associated with tryptophan depletion in mice, and tryptophan supplementation restores memory, as well as the expression of BDNF, NGF, and phosphorylated TrkB and the levels of Glo-1 and Glo-2 [15]. Whether tryptophan derived from *B. longum* Z1 links the reduction in MGO to the neurotrophic changes observed here remains to be tested. Since the liver is a major site of MGO metabolism, enhanced hepatic detoxification, if present at these doses, may lower circulating MGO levels, thereby reducing its delivery to the brain. This is relevant to cognition because elevated MGO levels are associated with cognitive decline and brain atrophy in older adults [18,19], experimental MGO exposure impairs memory in mice [15,23], and MGO suppresses the expression of neurotrophic factors in neurons [20]. Whether the lower MGO levels contributed to the neurotrophic or behavioral changes observed here, however, was not tested. The reduction in serum MGO by piracetam, despite its lack of effect on hepatic or brain MGO levels, may reflect effects on the peripheral handling of MGO, such as altered renal clearance or extracellular scavenging, rather than changes in hepatic or brain detoxification; this possibility warrants further investigation. It should also be noted that MGO has physiological roles. For example, it acts as a GABA_A_ receptor agonist, whereas Glo-1 overexpression has been shown to increase anxiety-like behavior in mice [33,34]. Therefore, the effects of long-term *B. longum* Z1 administration on emotional behavior should be evaluated.

This study had several limitations. First, the treatment period was short (7 days), only young, healthy, male mice were used, and the sample size for Western blotting and histology was small (*n* = 3), with animals selected by NORT performance rather than at random; the findings should be verified in models of cognitive impairment and in both sexes. No a priori sample-size calculation was performed, so non-significant results, such as those for BDNF, p-TrkA, p-AKT, and p-ERK, cannot be interpreted as evidence of no effect. Second, one animal per group was excluded from the original analysis without a pre-specified criterion; all available animals were included in the NORT analysis, but the tissues of these four animals were not processed. Third, the analyses were not performed blind to treatment, so observer bias cannot be fully excluded; however, NORT exploration was quantified automatically using identical settings for all animals. Hippocampal neurons were counted in a single section per animal without stereological sampling. Fourth, hepatic Nrf-2/glyoxalase signaling is reported only for a separate 100 mg/kg cohort, and fraction purity was not verified. In addition, ladder images were unavailable, the molecular forms of the NGF- and BDNF-immunoreactive bands were not confirmed, and loading controls and total proteins were detected on separate gels, so the Western blot data are semi-quantitative. Fifth, only a single batch of the preparation was tested, and inactivation was not confirmed by plating, so residual viable cells cannot be excluded; the unwashed preparation may have contained residual medium constituents, and safety was assessed only by ALT, AST, and hepatic and intestinal histology after 7 days at 5 and 20 mg/kg. Finally, causal links between MGO reduction, neurotrophic signaling, and memory were not tested (e.g., by TrkB or Glo-1 inhibition), and gut microbiota, barrier function, and microbial metabolites were not assessed [1].

## 4. Materials and Methods

### 4.1. Preparation of Heat-Inactivated B. longum Z1

*B. longum* Z1 was isolated from human feces and identified by 16S rRNA gene sequencing, as previously described [25], and deposited in the Korean Culture Center of Microorganisms (KCCM, Seoul, Republic of Korea) under accession number KCCM12660P. The strain was anaerobically cultured in Gifu Anaerobic Medium (GAM; Nissui Pharmaceutical, Tokyo, Japan) at 37 °C for three days and harvested by centrifugation. The cell pellet was freeze-dried without washing, resuspended in phosphate-buffered saline (pH 7.4), and heat-inactivated at 80 °C for 10 min; the inactivated cells were stored at −80 °C until use. Loss of viability was not confirmed by plating, and because the cells were not washed, the preparation may have contained residual medium constituents. Doses were defined as the dry weight of the freeze-dried preparation; based on a previously established conversion (1 mg ≈ 5 × 10^7^ CFU before inactivation) [25], 5, 20, and 100 mg/kg corresponded to approximately 2.5 × 10^8^, 1 × 10^9^, and 5 × 10^9^ CFU-equivalents/kg, respectively. A single batch of the preparation, identical to that used in our previous study [25], was used for all experiments; batch-to-batch consistency was not assessed. For oral administration, the preparation was suspended in 1% dextrose. Hereafter, *B. longum* Z1 refers to this heat-inactivated preparation.

### 4.2. Animals and Experimental Design

Male C57BL/6 mice (6 weeks old) were purchased from Orient Bio (Seongnam, Republic of Korea) and housed three or four mice per cage in standard polycarbonate cages with bedding under controlled conditions (22 ± 2 °C, 50 ± 10% relative humidity, 12 h light/dark cycle) with free access to standard chow and water; no additional environmental enrichment was provided. After seven days of acclimatization, the mice were randomly assigned to four groups (*n* = 7 per group) after stratification by body weight: Normal (1% dextrose), PC (piracetam 400 mg/kg in 1% dextrose), Low-Z1 (*B. longum* Z1 5 mg/kg in 1% dextrose), and High-Z1 (*B. longum* Z1 20 mg/kg in 1% dextrose). The experimental unit was the individual mouse. Treatments were administered by oral gavage once daily for seven days at a volume of 10 mL/kg. Animals were checked daily for general condition, and body weight was recorded during the treatment period. No specific humane endpoints were predefined because the study involved only short-term oral administration and non-invasive behavioral tests; no adverse events were observed.

The Y-maze test was performed on the day after the final administration, and the NORT was performed over the following two days (Figure 1A). No inclusion or exclusion criteria were set a priori. All available animals were included in the NORT analysis (*n* = 7 per group). In the original analysis, one mouse per group had been excluded after data inspection on the basis of the Y-maze test (in the Normal and PC groups, the recording was of insufficient quality to be scored; in the Low-Z1 and High-Z1 groups, spontaneous alternation was markedly lower than in the other animals [30.35% and 30.50%]); these exclusions were not based on a pre-specified criterion, and when Grubbs’ test (two-sided, α = 0.05) was applied post hoc to the spontaneous alternation values, only the High-Z1 value met the outlier criterion. The results of the analysis after exclusions are provided in Supplementary Table S2. Tissues from these four animals had not been processed, so tissue analyses were performed on the remaining six mice per group.

After completion of the NORT, mice were anesthetized by inhalation of isoflurane (Ifran; Hana Pharm, Seoul, Republic of Korea), and a surgical plane of anesthesia was confirmed by loss of the pedal withdrawal reflex. Blood was collected by cardiac puncture, and the mice were euthanized by exsanguination; death was confirmed by cessation of heartbeat and respiration. The brain, liver, small intestine, and large intestine were then excised. Serum was obtained by centrifugation (12,000 rpm, 15 min, 4 °C). Each brain was divided into hemispheres: one was stored at −80 °C for Western blotting and MGO measurement, and the other was fixed in 4% paraformaldehyde for histology. Other tissues for biochemical analyses were stored at −80 °C, and those for histology were fixed in 4% paraformaldehyde. Animals for tissue analyses were selected after behavioral testing on the basis of NORT performance, as described in Section 4.5, Section 4.6, and Section 4.8 and Supplementary Table S3.

In a separate exploratory experiment to examine whether *B. longum* Z1 activates hepatic Nrf-2/glyoxalase signaling in vivo, male C57BL/6 mice were administered 1% dextrose (Normal, *n* = 4) or *B. longum* Z1 at 100 mg/kg in 1% dextrose (*n* = 5) by oral gavage once daily for seven days under the same conditions. A higher dose than in the main study was used to increase the likelihood of detecting a pathway response in healthy mice. Liver tissues were collected 16 h after the final administration using the same anesthetic and terminal procedures, and no animals were excluded.

Both experiments, including all doses administered, were approved by the Institutional Animal Care and Use Committee of Gachon University (LCDI-2019-0181; date of approval: 8 January 2020).

### 4.3. Y-Maze Test

A Y-maze spontaneous alternation test was also performed on the day after the final administration [15,35]. Because the arm-entry data could not be verified against the original records, the Y-maze results are not reported.

### 4.4. Novel Object Recognition Test

NORT was conducted using previously described methods with minor modifications [15,36]. On the day before training, each mouse was habituated to the empty open-field box (45 cm × 45 cm × 45 cm) for 10 min. During training, each mouse explored two identical objects in the box for 3 min. After 24 h, one familiar object was replaced with a novel target object, and exploration was recorded for 3 min using SMART software (version 3.0; Panlab, Barcelona, Spain). The familiar and novel objects were glass bottles of identical size and shape that differed only in color (red vs. yellow). The objects were fixed to the floor of the box so that they could not be moved by the mice. The identity and position of the novel object were counterbalanced within each group. Exploration was defined as the presence of the animal’s head within a 1-cm zone around an object, as detected automatically by SMART 3.0; time spent climbing on an object could not be distinguished from active exploration. Exploration time was quantified automatically with identical zone settings for all animals; scoring was not blinded to treatment. No minimum-exploration criterion was applied. The box and objects were cleaned between trials to remove olfactory cues. The recognition index (%) was calculated as [novel-object exploration time/(novel-object exploration time + familiar-object exploration time)] × 100. Familiar, novel, and total exploration times for each animal are provided in Supplementary Table S4.

### 4.5. Western Blot Analysis

Brain hemispheres and liver tissues were homogenized in radioimmunoprecipitation assay buffer containing protease and phosphatase inhibitors, followed by centrifugation. Nuclear and cytosolic fractions of the liver were prepared using NE-PER Nuclear and Cytoplasmic Extraction Reagents (Thermo Fisher Scientific, Waltham, MA, USA) according to the manufacturer’s instructions. The three mice per group with the highest NORT recognition index were selected after the behavioral tests; selection was not random, and the Western blot data are therefore exploratory. Each lane represents one animal. Protein concentrations were determined using the Bradford assay, and equal amounts of protein (30–50 μg) were separated by sodium dodecyl sulfate-polyacrylamide gel electrophoresis and transferred onto polyvinylidene fluoride membranes using a Trans-Blot Turbo transfer system (Bio-Rad, Hercules, CA, USA). After blocking with 5% skim milk for 1 h at room temperature, the membranes were incubated overnight at 4 °C with primary antibodies and then with horseradish peroxidase-conjugated secondary antibodies for 2 h at room temperature. Each target protein was detected on a separate gel; all gels for a given figure were loaded with the same lysates in the same lane order, and the phosphorylated and total forms of each protein were detected on separate gels run in parallel. Membranes for TrkA, p-TrkA, p-AKT and Keap1 were cut horizontally before antibody incubation; no membrane was stripped and reprobed. A pre-stained protein ladder (PageRuler Prestained Protein Ladder, Thermo Fisher Scientific, Waltham, MA, USA) was loaded on every gel, but images of the ladder could not be acquired because of an imaging-system malfunction; band identity was therefore assigned from the ladder bands visible on the membrane and from the expected molecular weight of each target. For NGF and BDNF, the immunoreactive bands at approximately 23 and 34 kDa were quantified; because the ladder could not be imaged and the molecular forms (precursor or mature) were not confirmed, these are referred to as NGF- and BDNF-immunoreactive bands; for TrkB, the lower band of the doublet was quantified. Original uncropped images are provided in the Supplementary Materials.

The following primary antibodies were used: p-TrkA (#9141), AKT (#9272), p-AKT (#9271), ERK1/2 (#9102), p-ERK1/2 (#9101), HO-1 (#70081), TrkA (#9142), TrkB (#4603), CREB (#9197), p-CREB (#9198), Keap1 (#8047), histone H3 (#9715), and α-tubulin (#2144) from Cell Signaling Technology (Danvers, MA, USA); Glo-1 (sc-133144), Glo-2 (sc-271663), and Nrf-2 (sc-365949) from Santa Cruz Biotechnology (Dallas, TX, USA); p-TrkB (ab229908) and NGF (ab66459) from Abcam (Cambridge, UK); and BDNF (A11028) from ABclonal (Woburn, MA, USA). Horseradish peroxidase-conjugated goat anti-rabbit IgG (H+L) (#31460) and goat anti-mouse IgG (H+L) (#31430) secondary antibodies (Invitrogen, Thermo Fisher Scientific, Waltham, MA, USA; 1:2000) were used (Supplementary Table S1). Immunoreactive bands were visualized using SuperSignal West Pico PLUS Chemiluminescent Substrate (Thermo Fisher Scientific, Waltham, MA, USA) and a ChemiDoc XRS+ imaging system (Bio-Rad, Hercules, CA, USA), and band intensities were quantified using ImageJ software (version 1.53; National Institutes of Health, Bethesda, MD, USA). Phosphorylated proteins were normalized to their corresponding total proteins, nuclear Nrf-2 was normalized to histone H3, and all other proteins were normalized to α-tubulin. Because the loading controls and total proteins were detected on separate gels loaded with the same lysates in the same lane order, this normalization does not correct for gel-to-gel differences in transfer or detection, and the quantification should be regarded as semi-quantitative. Data are expressed as fold change relative to the Normal group. Brain samples were analyzed in three mice per group, and in the additional high-dose experiment, hepatic proteins were compared between the Normal (*n* = 4) and *B. longum* Z1 (100 mg/kg; *n* = 5) groups.

### 4.6. Histological Analysis

Histological analysis was performed as previously described [15]. Brain, liver, small intestine, and large intestine tissues fixed in 4% paraformaldehyde were dehydrated in graded ethanol, cleared in xylene, embedded in paraffin, and sectioned at 5 μm thickness. The sections were stained using a hematoxylin and eosin (H&E) staining kit (Sigma-Aldrich, St. Louis, MO, USA) and observed at 4× and 20× magnification using a Nikon Eclipse 80i microscope (Nikon, Tokyo, Japan). Histology was performed on the same three mice per group used for western blotting, using the contralateral brain hemisphere. For each animal, one coronal section through the dorsal hippocampus, in which the CA1, CA3, and DG subregions were clearly identifiable, was analyzed. Neurons were counted in three non-overlapping 20× fields per region within the pyramidal cell layers of CA1 and CA3 and the granule cell layer of the DG using ImageJ software (version 1.53; National Institutes of Health, Bethesda, MD, USA). Neurons were identified by their round nuclei with a visible nucleolus and clearly defined cell boundaries; small, darkly stained glial nuclei and shrunken or pyknotic cells were excluded. The three field counts were averaged to give one value per animal, which was used for statistical analysis (*n* = 3 per group). The outcome is therefore expressed as the number of neurons per 20× field and does not represent neuronal density or total neuronal number. Counting was not blinded to treatment. Liver and intestinal sections were assessed qualitatively.

### 4.7. Serum Biochemical Analysis

Serum ALT and AST activities were measured using commercial assay kits (Asan Pharmaceutical, Seoul, Republic of Korea), according to the manufacturer’s instructions.

### 4.8. Determination of Methylglyoxal by HPLC

MGO was quantified as its 2-methylquinoxaline (2-MQ) derivative after derivatization with *o*-phenylenediamine. Sample deproteinization and derivatization were based on the method of Chaplen et al. [37], and chromatographic quantification using 5-methylquinoxaline as an internal standard with UV detection at 315 nm was based on Chayaratanasin et al. [38]. The method was modified for tissue and serum samples, with derivatization for 24 h in the dark and isocratic separation using acetonitrile/water (20:80, *v*/*v*). Briefly, tissue homogenates and serum were deproteinized with 0.1 N perchloric acid, spiked with 5-methylquinoxaline (5-MQ; Sigma-Aldrich, St. Louis, MO, USA) as an internal standard, and derivatized with *o*-phenylenediamine at room temperature for 24 h in the dark. The derivatives were analyzed using a Waters e2695 Separation Module (Waters Corp., Milford, MA, USA) equipped with a Kromasil C18 column (150 mm × 4.6 mm, 5 μm; Nouryon, Bohus, Sweden) and a UV detector set at 315 nm. Separation was performed isocratically using acetonitrile/water (20:80, *v*/*v*) as the mobile phase at a flow rate of 1.0 mL/min. MGO concentrations were calculated from the peak-area ratio of 2-MQ to 5-MQ using a 2-MQ standard curve and were expressed as pmol/g tissue for the liver and brain and pmol/mL for serum. Representative chromatograms of the liver, brain, and serum samples are presented in Figures S2–S4, where the 2-MQ and 5-MQ peaks were well resolved from endogenous interfering peaks in all matrices. Serum and liver MGO were measured in the six mice per group whose tissues had been processed (Section 4.2). Brain MGO was measured in the four mice per group with the highest NORT recognition index. Each sample was measured once.

### 4.9. Statistical Analysis

Animals were randomly assigned to treatment groups after stratification by body weight, whereas animals for tissue analyses were selected by NORT performance (Supplementary Table S3). No formal sample-size calculation was performed; the group size was based on our previous study using the same behavioral tests [15]. No primary outcome was pre-specified; the NORT recognition index is the outcome on which the main conclusion is based. Behavioral testing, histological counting, and tissue analyses were not performed blind to treatment. The NORT data were analyzed using all available animals; the original analysis after post hoc exclusion of one animal per group is reported as a sensitivity analysis (Supplementary Table S2). No correction for multiple comparisons was applied across endpoints or across the proteins examined by Western blotting; non-significant trends are therefore reported as hypothesis-generating only. Data are presented as mean ± standard error of the mean (SEM). Statistical significance was determined by one-way analysis of variance (ANOVA) followed by Tukey’s post hoc test for comparisons among the four groups and by an unpaired two-tailed Student’s *t*-test for comparisons between two groups (Figure S1) using GraphPad Prism (version 5.0; GraphPad Software, San Diego, CA, USA). In the NORT, novel- and familiar-object exploration times within each group were compared using paired two-tailed *t*-tests. A *p*-value < 0.05 was considered statistically significant. Statistical calculations for the MGO and NORT exploration data were additionally verified using Claude (Claude Opus 5.5; Anthropic, San Francisco, CA, USA), with the same raw data as the primary analyses, which were performed using GraphPad Prism; the results were concordant. Sensitivity analyses of the NORT data and Grubbs’ tests on the NORT recognition index (Supplementary Table S2) were computed in Python (version 3.13; Python Software Foundation, Wilmington, DE, USA) using the SciPy library (version 1.18).

## 5. Conclusions

Oral administration of heat-inactivated *B. longum* Z1 improved recognition memory in normal mice, with no significant changes in serum liver enzymes or apparent hepatic or intestinal histological abnormalities after 7 days of administration at the doses tested. In exploratory analyses of mice selected by NORT performance, this effect was accompanied by higher levels of an NGF-immunoreactive band, higher TrkB and CREB phosphorylation, and a higher granule cell count per field in the DG; MGO levels were lower in the liver, brain, and serum, and in a separate cohort, a higher dose (100 mg/kg) increased hepatic Glo-1, Glo-2, and nuclear Nrf-2 levels. Although these findings are associative and do not establish that neurotrophic signaling or MGO reduction mediates the behavioral effect, they suggest that the heat-inactivated *B. longum* Z1 preparation tested here (a single, unwashed batch whose inactivation was not confirmed by plating) is a potential postbiotic candidate for supporting cognitive function. Further studies using pathway inhibition, cognitive impairment models, and human trials are warranted.

## 6. Patents

Kim, S.Y.; Kim, D.-H.; Baek, H.-M.; Hong, S.-M. Novel *Bifidobacterium longum* Z1 Strain and Use Thereof. Korean Patent No. 10-2396476, 4 May 2022.

## Data Availability

The data presented in this study are available in the article and its Supplementary Materials.

## Supplementary Materials

The following supporting information can be downloaded at: https://www.mdpi.com/article/10.3390/ijms272110054/s1.

## Abbreviations

The following abbreviations are used in this manuscript:

2-MQ	2-Methylquinoxaline
5-MQ	5-Methylquinoxaline
AKT	Protein kinase B
ALT	Alanine aminotransferase
ANOVA	Analysis of variance
ARE	Antioxidant response element
AST	Aspartate aminotransferase
BDNF	Brain-derived neurotrophic factor
CA	Cornu ammonis
CREB	cAMP response element-binding protein
DG	Dentate gyrus
ERK	Extracellular signal-regulated kinase
Glo-1	Glyoxalase-I
Glo-2	Glyoxalase-II
H&E	Hematoxylin and eosin
HO-1	Heme oxygenase-1
HPLC	High-performance liquid chromatography
Keap1	Kelch-like ECH-associated protein 1
MAPK	Mitogen-activated protein kinase
MGO	Methylglyoxal
NGF	Nerve growth factor
NORT	Novel object recognition test
Nrf-2	Nuclear factor erythroid 2-related factor 2
PC	Positive control
PI3K	Phosphatidylinositol 3-kinase
SEM	Standard error of the mean
TrkA	Tropomyosin receptor kinase A
TrkB	Tropomyosin receptor kinase B
