# Supplementary material for: Heat-Inactivated Bifidobacterium longum Z1 Improves Recognition Memory in Mice in Association with Enhanced Trk-CREB Signaling and Reduced Methylglyoxal Levels
Source: Int J Mol Sci. 2026 Nov 9;27(21):10054. doi: 10.3390/ijms272110054 (PMC13764283; doi:10.3390/ijms272110054)
Supplement: Supplementary file 1 [file ijms-27-10054-s001.zip › Z1_IJMS_Supplementary_Materials_clean.pdf]

Supplementary Materials

Heat-Inactivated *Bifidobacterium longum* Z1 Improves Recognition Memory in Mice in Association with Enhanced Trk-CREB Signaling and Reduced Methylglyoxal Levels

Seong-Min Hong [1,†], Jaehoon Bae[2,3,†], Seojin Kang [1], Hyunjin Kang [1], Dong-Hyun Kim [4,*] and Sun Yeou Kim [5,*]

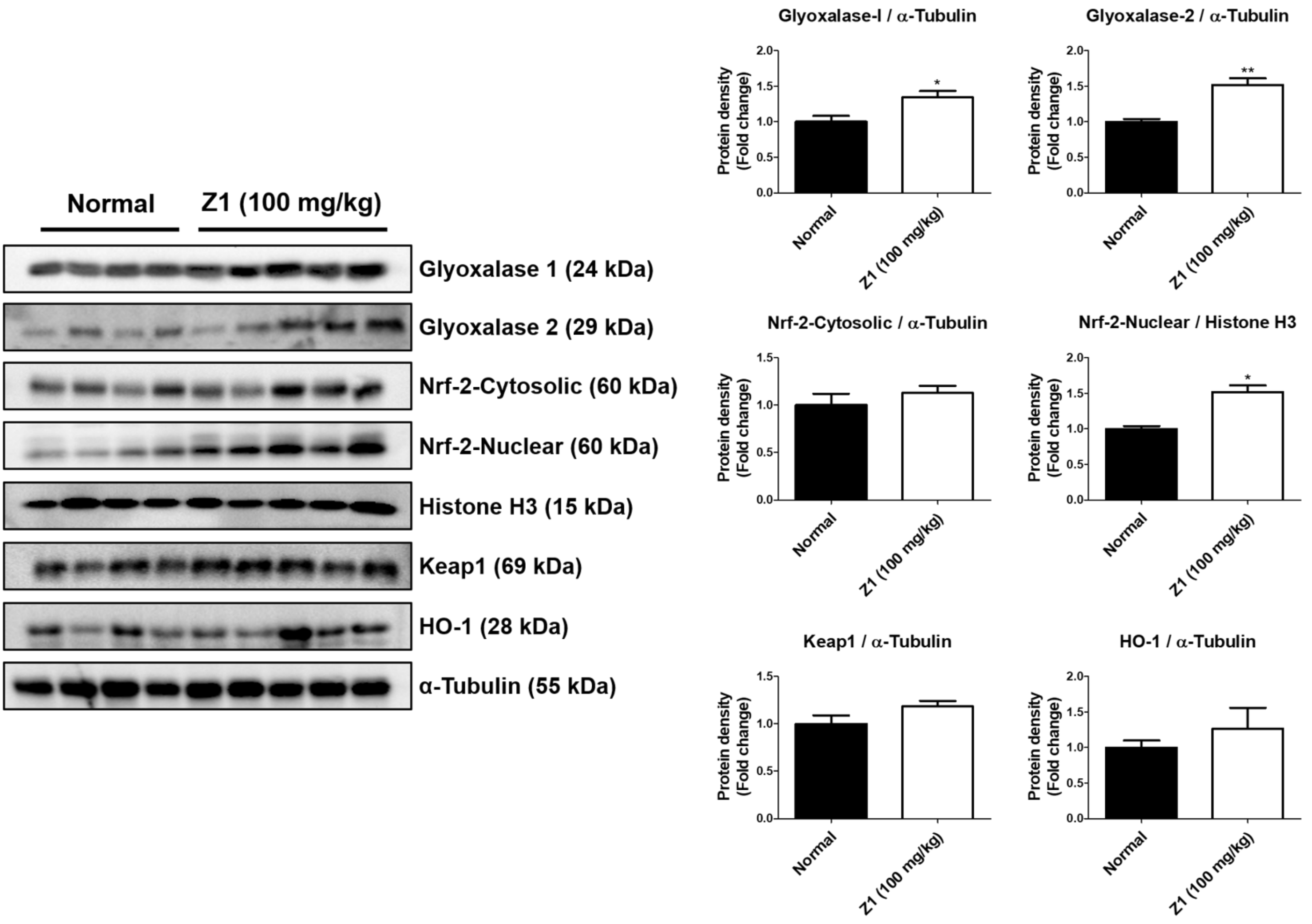

Figure S1. Effects of a high dose of *B. longum* Z1 (100 mg/kg) on hepatic Nrf-2 signaling and the glyoxalase system. Mice were orally administered 1% dextrose (Normal, $n = 4$) or *B. longum* Z1 (100 mg/kg, $n = 5$) once daily for 7 days. Representative Western blots and densitometric quantification of glyoxalase-I, glyoxalase-II, cytosolic and nuclear Nrf-2, Keap1, and HO-1 in the liver. α-Tubulin and histone H3 were used as loading controls for cytosolic/total and nuclear proteins, respectively. Data are presented as mean ± SEM. $^{*}p < 0.05$ and $^{**}p < 0.01$ vs. Normal group (Student's *t*-test). Data were obtained from a separate cohort independent of the main behavioral study; liver tissues were collected 16 h after the final administration.

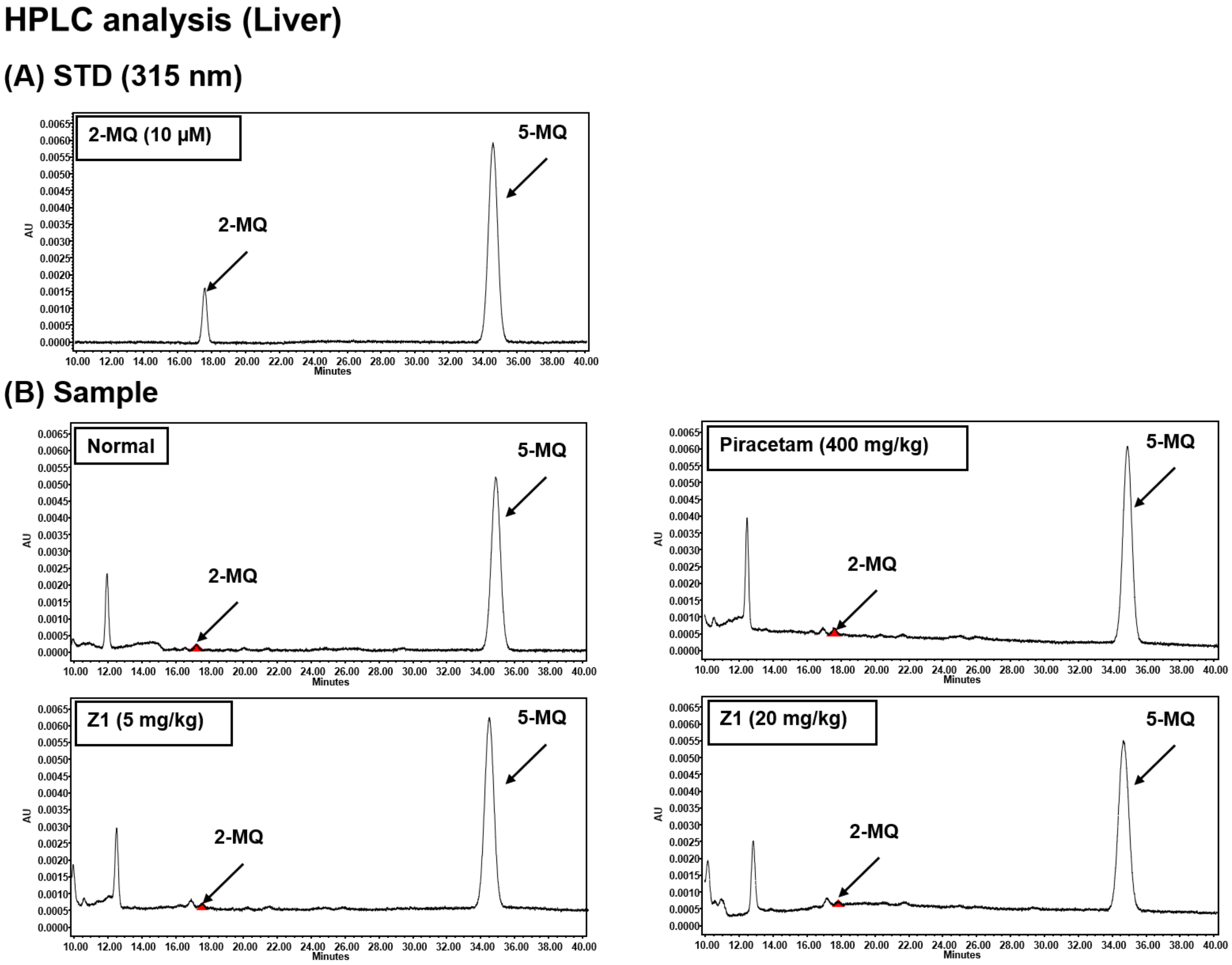

Figure S2. Representative HPLC chromatograms for methylglyoxal quantification in liver tissues. (**A**) Standard chromatogram (315 nm) showing 2-methylquinoxaline (2-MQ, 10 μM) and 5-methylquinoxaline (5-MQ, internal standard) peaks obtained after *o*-phenylenediamine derivatization of methylglyoxal. (**B**) Representative chromatograms of liver samples from the Normal, PC, Low-Z1, and High-Z1 groups.

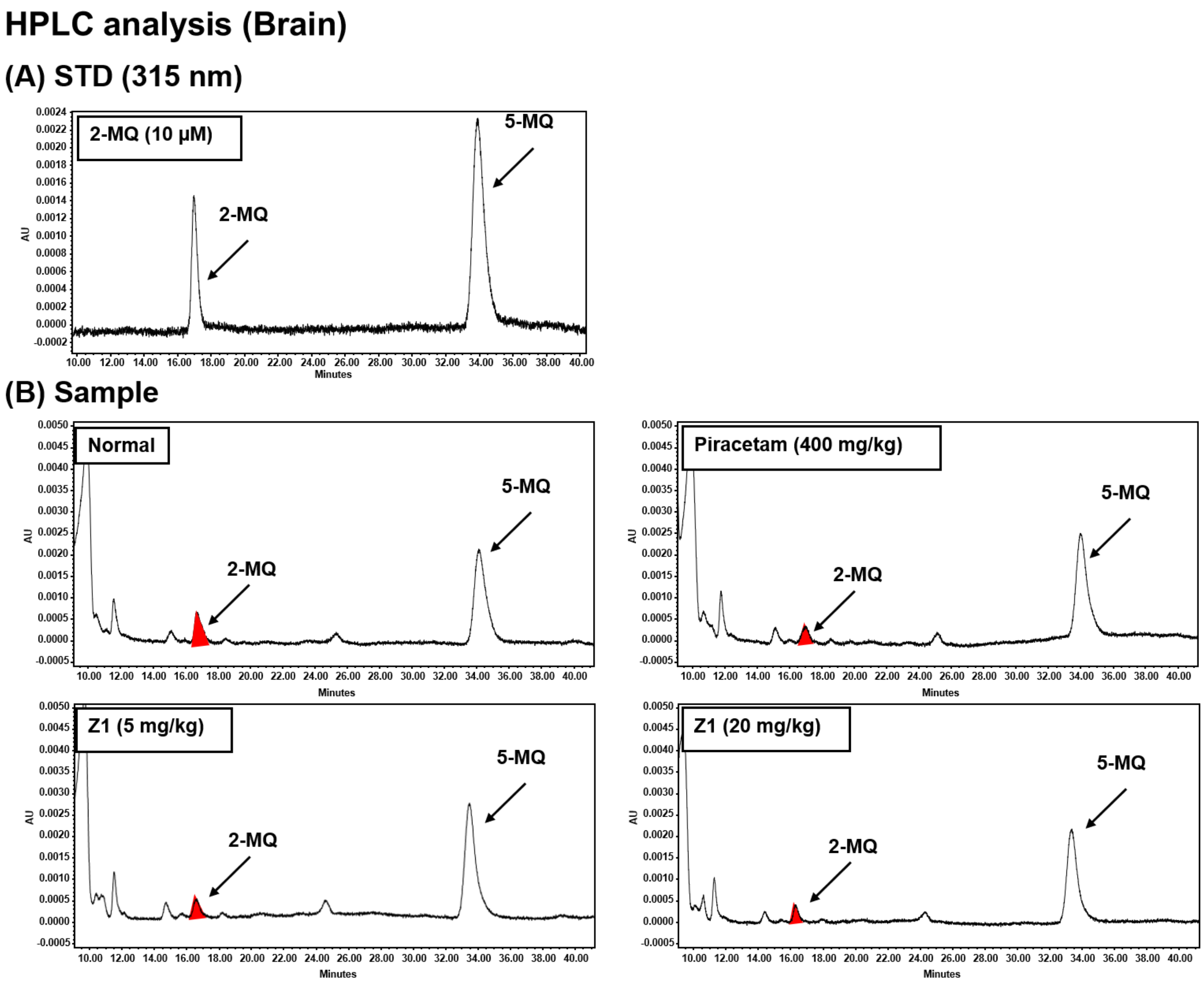

Figure S3. Representative HPLC chromatograms for methylglyoxal quantification in brain tissues. (**A**) Standard chromatogram (315 nm) showing 2-methylquinoxaline (2-MQ, 10 μM) and 5-methylquinoxaline (5-MQ, internal standard) peaks obtained after *o*-phenylenediamine derivatization of methylglyoxal. (**B**) Representative chromatograms of brain samples from the Normal, PC, Low-Z1, and High-Z1 groups.

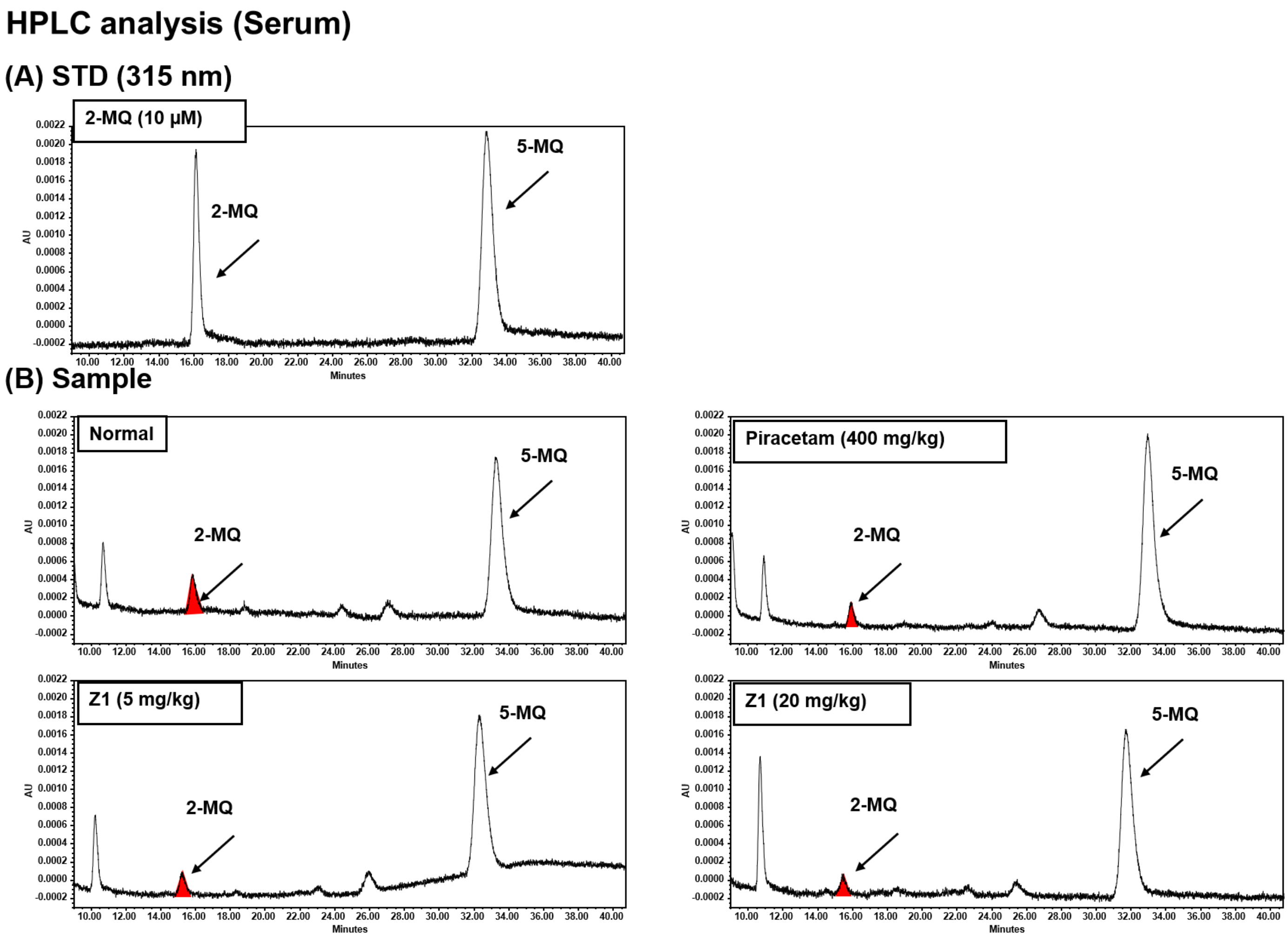

Figure S4. Representative HPLC chromatograms for methylglyoxal quantification in serum. (**A**) Standard chromatogram (315 nm) showing 2-methylquinoxaline (2-MQ, 10 μM) and 5-methylquinoxaline (5-MQ, internal standard) peaks obtained after *o*-phenylenediamine derivatization of methylglyoxal. (**B**) Representative chromatograms of serum samples from the Normal, PC, Low-Z1, and High-Z1 groups.
